# Pulmonary *Tropheryma whipplei* Infection Presenting With Multiple Thick‐Walled Cavities on Chest CT: A Case Report and Literature Review

**DOI:** 10.1002/rcr2.70487

**Published:** 2026-01-30

**Authors:** Yang Liu, Handan Fu

**Affiliations:** ^1^ Respiratory Department Shaoxing City Keqiao District Hospital of Traditional Chinese Medicine Shaoxing China

**Keywords:** chest CT, metagenomic next‐generation sequencing, pneumonia, thick‐walled cavities, *Tropheryma whipplei*

## Abstract

Whipple's disease (WD) is a rare chronic multisystem infectious disease caused by the actinomycete *Tropheryma whipplei* . Pulmonary involvement is uncommon, and its clinical manifestations lack specificity, with diverse imaging findings, making it prone to misdiagnosis. We report a rare case of a 50‐year‐old woman who presented with a 2‐week history of cough. Chest CT showed multiple thick‐walled cavities in both lungs, a highly unusual presentation for WD pneumonia. Routine microbiological tests, including acid‐fast staining and culture of bronchoalveolar lavage fluid (BALF), were negative, which made the diagnosis challenging. Metagenomic next‐generation sequencing (mNGS) of BALF detected *T. whipplei* , confirming the diagnosis of WD pneumonia. After oral doxycycline treatment, follow‐up chest CT showed complete resolution of the pulmonary cavities. This case demonstrates that multiple thick‐walled cavities may be a characteristic imaging manifestation of WD pneumonia, highlights the diagnostic value of mNGS for this rare infection, and supports oral doxycycline monotherapy as an effective treatment option for isolated pulmonary TW infection.

## Graphical abstract

This case report highlights a rare instance of pulmonary infection caused by *Tropheryma whipplei* , presenting with distinctive imaging features on chest CT: multiple round or oval thick‐walled consolidations with small central cavities (approximately 1 mm in diameter) and subpleural distribution. The diagnosis was confirmed through metagenomic next‐generation sequencing of bronchoalveolar lavage fluid, and effective treatment was achieved with oral doxycycline.

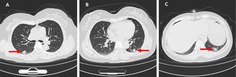

## 1 Introduction

Whipple's disease (WD) is a chronic multisystem infectious disease caused by the actinomycete *Tropheryma whipplei* (TW) [1]. WD can affect multiple organ systems, including the gastrointestinal tract, joints, central nervous system, cardiovascular system, and lungs [2]. Traditionally, WD was considered an intestinal disorder; however, recent evidence indicates that pulmonary involvement occurs more frequently than previously recognised [3, 4, 5]. However, the clinical manifestations of WD pneumonia lack specificity, and its imaging findings are diverse, making it prone to misdiagnosis [6]. With the application of metagenomic next‐generation sequencing (mNGS) in clinical practice, reports of TW pneumonia cases have gradually increased [6, 7, 8, 9], but there is no consensus on its diagnostic criteria, treatment regimens, and efficacy evaluation. Therefore, raising awareness of WD pneumonia and employing sensitive diagnostic techniques and appropriate treatment strategies are crucial.

This article reports a rare case of WD pneumonia presenting with multiple thick‐walled cavities on chest CT, aiming to raise clinicians' awareness of the disease, explore the role of mNGS in diagnosis, and discuss treatment options.

## 2 Case Report

A 50‐year‐old woman presented with a 2‐week history of cough on March 24, 2025. The cough was non‐productive, not associated with fever, chest pain, hemoptysis, or dyspnea. She had no significant past medical history and denied any history of chronic diarrhea, abdominal pain, weight loss, joint pain, arthritis, headache, nausea, vomiting, or limb weakness. She had no known exposure to sewage or soil. The patient had not received any antibiotic therapy prior to presentation.

On admission, vital signs were: temperature 37°C, blood pressure 132/87 mmHg, heart rate 96 beats/min, respiratory rate 20 breaths/min, and oxygen saturation 98% on room air. Physical examination revealed: lungs—clear breath sounds bilaterally with no crackles, wheezes, or rhonchi; cardiovascular—regular heart rhythm with no murmurs; abdomen—soft, non‐tender, no organomegaly; musculoskeletal—no joint swelling or tenderness; neurological—normal mental status, cranial nerves, motor and sensory function.

Laboratory tests showed a white blood cell count of 8.0 × 10^9^/L with 82.2% neutrophils, C‐reactive protein of 37.0 mg/L, and D‐dimer of 1.29 mg/L. Liver and kidney function, immunoglobulins, CD4 and CD8 T‐lymphocyte counts, and antinuclear antibodies were within normal limits. Sputum smear for acid‐fast bacilli and sputum culture were negative.

**FIGURE 1:**
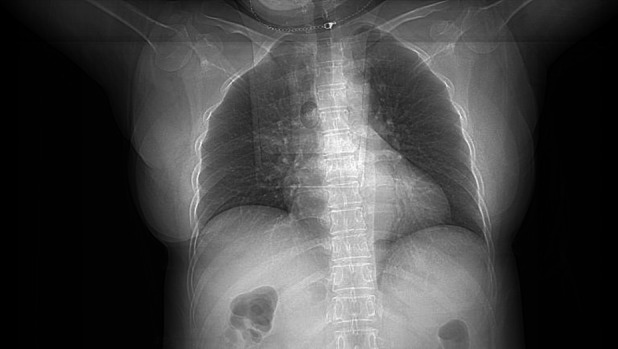
Chest radiograph (CXR) performed on March 24, 2025 showed no obvious abnormalities and no visible infiltrates, cavities, or masses.

**FIGURE 2:**
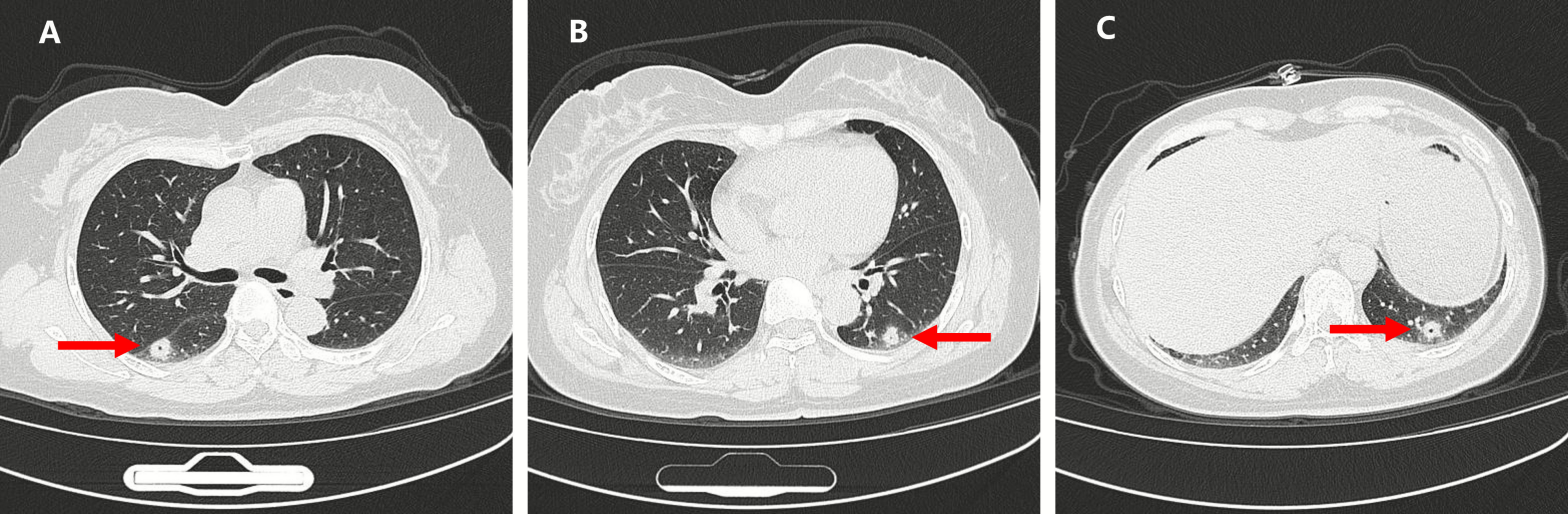
Chest CT on March 24, 2025, showing multiple subpleural thick‐walled cavities in both lungs (red arrows). The cavities demonstrated distinctive features: Round or oval consolidations with thick walls, small central cavities (approximately 1 mm in diameter) and characteristic subpleural distribution, predominantly located in the lower lobes.

**FIGURE 3:**
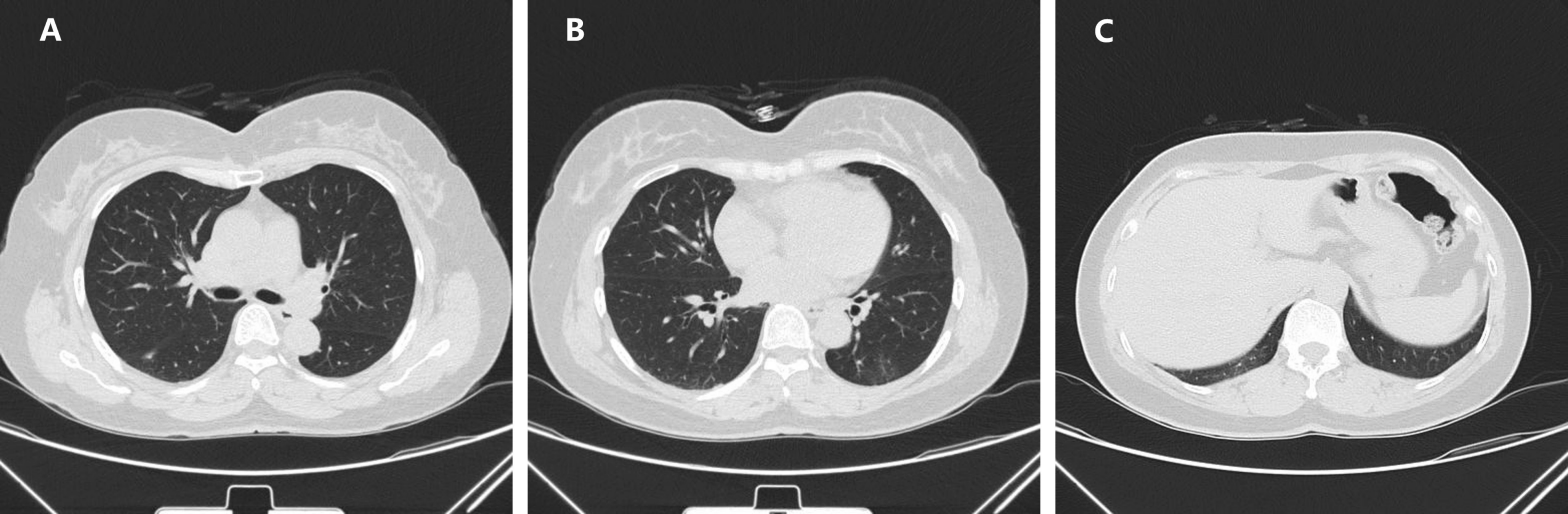
Follow‐up chest CT on May 20, 2025, showing resolution of the cavities in both lungs.

Chest radiograph (CXR) performed on admission showed no obvious abnormalities, with clear lung fields and no visible infiltrates, cavities, or masses (Figure 1). Chest CT showed multiple thick‐walled cavities in both lungs (Figure 2). All cavities shared similar characteristics: they were predominantly located in the lower lobes, abutting the pleural surface in a peripheral distribution. Each lesion appeared as a round or oval mass with thick walls, featuring a small central cavity (approximately 1 mm in diameter) surrounded by pulmonary consolidation. Based on the imaging findings of multiple thick‐walled cavities, the initial differential diagnoses included pulmonary tuberculosis with cavitation, fungal pneumonia, bacterial lung abscess, and infection caused by other uncommon pathogens. Empirical antibiotic therapy with levofloxacin 0.5 g IV once daily was initiated on admission. Bronchoscopy revealed no abnormalities in the tracheal and bronchial mucosa. Given the negative sputum culture results and high suspicion for infection with uncommon or fastidious pathogens, both conventional BALF culture and mNGS were performed simultaneously after discussion with the patient's family. This approach was chosen to facilitate early pathogen identification and avoid the need for a second bronchoscopy if conventional cultures remained negative. BALF routine culture and acid‐fast staining were negative. BALF mNGS detected 40,625 sequences of *T. whipplei* . Based on these findings, a diagnosis of *T. whipplei* pneumonia was made. To assess for potential systemic involvement of Whipple's disease, comprehensive evaluation was performed. The patient had no gastrointestinal, cardiovascular, musculoskeletal, or neurological symptoms. Abdominal ultrasonography and echocardiography were unremarkable. Physical examination revealed no abdominal tenderness, organomegaly, arthritis, or neurological deficits. Based on this evaluation, there was no evidence of extrapulmonary involvement, confirming the diagnosis of isolated pulmonary *T. whipplei* infection. After the diagnosis of *T. whipplei* pneumonia was confirmed, levofloxacin was discontinued and the patient was started on oral doxycycline 100 mg twice daily for 2 months. The cough significantly improved after 2 weeks of treatment and completely resolved after 4 weeks. A follow‐up chest CT 2 months after treatment (May 20, 2025) showed resolution of the cavities in both lungs (Figure 3). The patient has been followed up regularly until November 2025 without recurrence of symptoms or radiological abnormalities.

## 3 Discussion

TW is a gram‐positive rod‐shaped bacterium, and patients with a history of sewage or soil contact and immunodeficiency are susceptible [1]. The latest research from the United States shows that the incidence of Whipple's disease is 4.6 per 1,000,000 hospitalised patients [10]. TW can involve multiple systems, such as the cardiovascular and central nervous systems, while respiratory system infections are relatively rare, accounting for about 4.1% of hospitalised patients with Whipple's disease [10]. The clinical manifestations of WD pneumonia lack specificity, mainly including chest pain, chronic cough, sputum production, and dyspnea, often accompanied by recurrent arthralgia or arthritis, endocarditis, chronic diarrhoea, abdominal pain, and weight loss [1, 2]. If not treated promptly, many serious complications can occur, and the prognosis is poor [11, 12, 13].

To date, there is no systematic description of the imaging findings of TW pneumonia. Previous case reports have shown that the imaging findings of TW pulmonary infection are diverse, including ground‐glass opacities, consolidation, cavities, lung abscesses, and pleural effusion [7, 9, 14, 15, 16, 17, 18]. To better characterise the imaging spectrum of TW pneumonia, we conducted a systematic literature search in PubMed, Web of Science, and Embase databases using the keywords “ *T. whipplei* ,” “Whipple's disease,” and “ *T. whipplei* pneumonia” for articles published between January 1, 2008, and May 31, 2025. The inclusion criteria were: (1) a definite diagnosis of *T. whipplei* pneumonia confirmed by pathology, PCR, or gene sequencing, with symptom improvement after antimicrobial treatment; (2) available pulmonary imaging data; (3) complete case information. A total of 28 case reports of *T. whipplei* pneumonia were retrieved. Among them, only three patients had pulmonary cavities. With the addition of our case, a total of four cases of TW pneumonia with pulmonary cavities were identified (Table 1). All four cases had thick‐walled cavities with a peripheral distribution. However, the three previously reported cases [16, 17, 18] each presented with a single cavity, whereas our patient had multiple thick‐walled cavities—a presentation not previously documented in the literature. Of particular note, the cavities in our case exhibited distinctive imaging features: round or oval consolidations with thick walls, small central cavities (approximately 1 mm in diameter), and a characteristic subpleural distribution. This unique imaging pattern may represent a characteristic radiological feature of TW pneumonia. This finding is helpful for the early recognition of TW pneumonia but still needs to be differentiated from metastatic tumours, hematogenous infections, and pulmonary vasculitis. As for the cause of thick‐walled cavities in this disease, it may be related to different stages of the disease process and the patient's immune function, but this hypothesis needs to be verified with more cases.

**TABLE 1:** Clinical characteristics of patients with WD pneumonia and pulmonary cavities.

Case	Author and year of publication	Sex/age (years)	Underlying diseases	Clinical presentation	Chest CT scan finding	Diagnostic methods	Treatment	Outcome
1	Zhang et al. 2021 [16]	M, 26	None	Chest pain	A thick‐walled cavity in the left upper lung	Balf mNGS	Ceftriaxone 2 g QD IV for 2 weeks, then TMP‐SMX 0.96 g BID PO	Cavity resolved at 6 weeks
2	Fang et al. 2023 [17]	F, 49	None	Cough and phlegm	A thick‐walled cavity in the left upper lobe	Balf mNGS	Piperacillin‐tazobactam, moxifloxacin (duration not provided)	Cavity decreased in size at 1 month
3	Shan et al. 2024 [18]	M, 41	Cryptococcus, treatment JAK inhibitor	Hacking cough	A small thick‐walled cavity in the left	Balf mNGS	Ceftriaxone 2 g QID IV for 14 days, then TMP‐SMX PO for 12 months; fluconazole 400 mg QID IV, then 300 mg QD PO	Cavity initially enlarged at 4 weeks, then decreased in size at 15 weeks, nearly resolved at 6 months
4	Our case	F, 50	None	Cough	Three thick‐walled cavities in both lungs	Balf mNGS	Doxycycline PO 100 mg BID for 2 months	Cavities disappeared at 2 months

*Note:* QID: four times daily; IV: intravenous; TMP‐SMX: trimethoprim‐sulfamethoxazole; PO: oral; QD: once daily; BID: twice daily.

The diagnosis of WD has always been challenging. *T. whipplei* is difficult to culture in vitro and requires cultivation in human fibroblast and macrophage cell lines [19, 20, 21]. Currently, the detection techniques for TW include PAS staining, qPCR, and gene sequencing (including 16S rRNA sequencing, 23S rRNA sequencing, and mNGS). Early diagnosis of WD was based on the presence of granular “foamy” macrophages with positive PAS staining in duodenal biopsy, but its specificity and sensitivity are poor [22]. In recent years, with the development of molecular biology techniques, PCR and mNGS methods have been gradually applied to the diagnosis of WD, greatly improving the sensitivity and specificity of diagnosis [6, 23]. All four cavitary cases were diagnosed using BALF mNGS (Table 1), highlighting the important role of this technique in detecting TW when conventional cultures are negative. In this case, sputum culture and BALF smear and culture were negative, and the presence of multiple pulmonary cavities raised high suspicion for infection with uncommon pathogens. Therefore, mNGS was performed as a second‐line test simultaneously with conventional BALF culture during bronchoscopy to facilitate early pathogen identification and avoid the need for repeat bronchoscopy. While conventional BALF culture and smear were negative, mNGS detected 40,625 sequences of *T. whipplei* , confirming the diagnosis of WD pneumonia. This case demonstrates that for patients with atypical pulmonary infections and high clinical suspicion for uncommon pathogens, judicious use of mNGS can enable rapid diagnosis and timely targeted therapy.

There is no consensus on the optimal treatment regimen for WD. For classic Whipple's disease, treatment strategies have evolved from intravenous penicillin and doxycycline to streptomycin/penicillin or ceftriaxone for 2 weeks, followed by oral SMZ‐TMP for 1–2 years [1]. Due to the risk of SMZ/TMP resistance and allergy, some scholars have proposed a regimen of doxycycline combined with hydroxychloroquine for 12–18 months, followed by lifelong doxycycline maintenance therapy [24]. The latest research has confirmed the effectiveness of this regimen [25]. However, WD pneumonia is an acute or focal infection, and SMZ or doxycycline monotherapy is often used, with an uncertain duration [6, 23, 26]. Review of the four cavitary cases (Table 1) reveals diverse treatment approaches. Our patient achieved complete cavity resolution with 2 months of oral doxycycline monotherapy, suggesting that doxycycline therapy may be a practical option for pulmonary cavitary TW infection, and the duration can be adjusted according to the patient's condition, but whether it can prevent recurrence still needs further follow‐up observation.

In conclusion, this article reports a rare case of WD pneumonia presenting with a distinctive imaging pattern: multiple round or oval thick‐walled consolidations with small central cavities and subpleural distribution in both lungs. This unique imaging pattern may represent a characteristic radiological feature of WD pneumonia, which is important for its early recognition. BALF mNGS played a key role in the diagnosis of this case and is expected to become the preferred diagnostic method for patients with suspected WD pneumonia. Oral doxycycline monotherapy is effective and can be used as a simple and effective treatment option for WD pneumonia.

## Author Contributions

**Yang Liu:** conceptualization, data collection, writing the original draft, and reviewing the manuscript. **Handan Fu:** supervision, data analysis, and finalising the manuscript for submission.

## Funding

The authors have nothing to report.

## Ethics Statement

This case report was conducted in accordance with ethical standards and guidelines.

## Consent

Informed consent for publication was obtained from the patient involved in this study using the form provided by the Journal. The patient provided specific written consent for the publication of this manuscript, ensuring that all personal information was anonymized to protect their privacy.

## Conflicts of Interest

The authors declare no conflicts of interest.

## Data Availability

The data that support the findings of this study are available on request from the corresponding author. The data are not publicly available due to privacy or ethical restrictions.
